# Challenges and Opportunities behind the Use of *Herbaria* in Paleogenomics Studies

**DOI:** 10.3390/plants12193452

**Published:** 2023-09-30

**Authors:** Simone Papalini, Valerio Di Vittori, Alice Pieri, Marina Allegrezza, Giulia Frascarelli, Laura Nanni, Elena Bitocchi, Elisa Bellucci, Tania Gioia, Luis Guasch Pereira, Karolina Susek, Maud Tenaillon, Kerstin Neumann, Roberto Papa

**Affiliations:** 1 Department of Agricultural, Food and Environmental Sciences, Marche Polytechnic University, 60131 Ancona, Italy; s.papalini@pm.univpm.it (S.P.); a.pieri@univpm.it (A.P.); m.allegrezza@univpm.it (M.A.); g.frascarelli@univpm.it (G.F.); l.nanni@univpm.it (L.N.); e.bitocchi@univpm.it (E.B.); e.bellucci@univpm.it (E.B.); 2 School of Agricultural, Forestry, Food and Environmental Sciences, University of Basilicata, 85100 Potenza, Italy; tania.gioia@unibas.it; 3 Spanish Plant Genetic Resources National Center, National Institute for Agricultural and Food Research and Technology (CRF-INIA-CSIC), 28805 Alcalá de Henares, Madrid, Spain; luis.guasch@inia.csic.es; 4 Legume Genomics Team, Institute of Plant Genetics, Polish Academy of Sciences, Strzeszynska 34, 60-479 Poznan, Poland; ksus@igr.poznan.pl; 5 Génétique Quantitative et Evolution–Le Moulon, Université Paris-Saclay, INRAE, CNRS, AgroParisTech, 91190 Gif-sur-Yvette, France; maud.tenaillon@inrae.fr; 6 Leibniz Institute of Plant Genetics and Crop Plant Research (IPK) Gatersleben, 06466 Seeland, Germany; neumannk@ipk-gatersleben.de

**Keywords:** plant genetic resources, population genomics, crop evolution, food legumes

## Abstract

Paleogenomics focuses on the recovery, manipulation, and analysis of ancient DNA (aDNA) from historical or long-dead organisms to reconstruct and analyze their genomes. The aDNA is commonly obtained from remains found in paleontological and archaeological sites, conserved in museums, and in other archival collections. *Herbarium* collections represent a great source of phenotypic and genotypic information, and their exploitation has allowed for inference and clarification of previously unsolved taxonomic and systematic relationships. Moreover, *herbarium* specimens offered a new source for studying phenological traits in plants and for disentangling biogeography and evolutionary scenarios of species. More recently, advances in molecular technologies went in parallel with the decreasing costs of next-generation sequencing (NGS) approaches, which paved the way to the utilization of aDNA for whole-genome studies. Although many studies have been carried out combining modern analytic techniques and ancient samples, such as *herbarium* specimens, this research field is still relatively unexplored due to the need for improving strategies for aDNA manipulation and exploitation from ancient samples. The higher susceptibility of aDNA to degradation and contamination during *herbarium* conservation and manipulation and the occurrence of biochemical postmortem damage can result in a more challenging reconstruction of the original DNA sequence. Here, we review the methodological approaches that have been developed for the exploitation of historical *herbarium* plant materials, such as best practices for aDNA extraction, amplification, and genotyping. We also focus on some strategies to overcome the main problems related to the utilization of *herbarium* specimens for their exploitation in plant evolutionary studies.

## 1. Introduction

### 1.1. History and State of the Art of Herbaria

**Figure 1:**
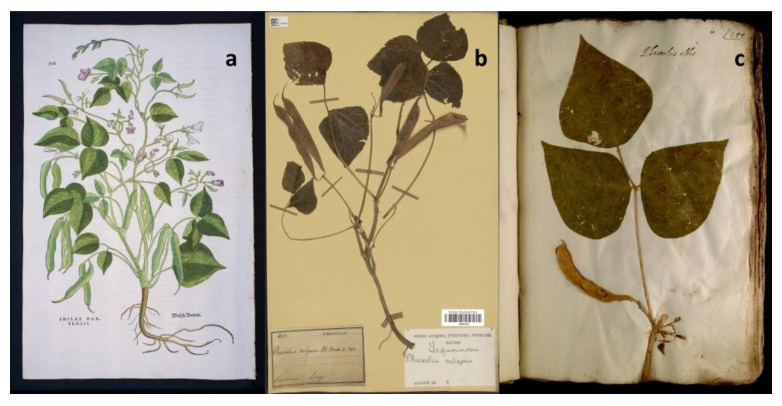
*Herbaria* illustrations. (**a**) The oldest illustration of a European common bean plant (*Phaseolus vulgaris*) from “*Di Historias Stirpium*” (Fuchs, 1542); “Courtesy of Hunt Institute for Botanical Documentation, Carnegie Mellon University, Pittsburgh, PA”. (**b**) *Phaseolus vulgaris* specimen from the Muséum National d’Histoire Naturelle (MNHN) (France), Paris, Collection: Vascular plants (P), Specimen P02872197, dated 1833. The label notes include information about the collector, the collection site, and the date. (**c**) *Phaseolus vulgaris* specimen from the *herbarium* of Ulisse Aldrovandi; “Courtesy of Alma Mater Studiorum University of Bologna—University Museum System—Herbarium and Botanical Garden”.

*Herbarium* collections represent large and not entirely explored deposits of genetic and phenotypic information [1] that are able to provide snapshots of the diversity that was present in the past. Initially, *herbaria* consisted of figurative books (Figure 1a) describing the characteristics of medicinal plants [2]. Over time, *herbaria* evolved to contain preserved plants, seeds, fungi, and algae collected at different phenological stages [3]. *Herbarium* specimens are commonly preserved or dried on paper sheets or stored in folded packets or small boxes, which can be treated with solvents or pesticides to protect the specimens. However, it is also possible to find plant material conserved in liquid solutions that may contain acids, aldehydes, alcohols, or other organic solvents for specimen preservation [4]. More recently, *herbarium* samples are usually provided with supplementary label notes, often including details about the collection site and observations made by the collector, as shown in Figure 1b [5]. The history of modern *herbaria* began in the 16th century, when Luca Ghini (1490–1556), a professor of medical botany at the University of Bologna and Pisa, developed a method to make plant specimens transportable and preservable over time [6,7]. However, his legacy mainly consists of books, manuscripts, and letters, since the dried plants collected by Ghini have been largely lost after his death. Ghini’s contribution to the field is highly recognized, also thanks to the *herbaria* established by his disciples. Some notable examples include the oldest surviving *herbarium*, the “*Erbario Cibo*”, preserved at the Pontifical University Library in Rome, and the *herbarium* of Ulisse Aldrovandi (1522–1605) that is conserved in Bologna (Figure 1c) [8,9,10,11]. In the 18th century, Carl Linnaeus developed his *herbarium* “cabinet” collection, including approximately 14,000 sheets of plant specimens and several zoological specimens [12]. In addition to his *herbaria*, Linnaeus provided innovative instruction on how to establish an *herbarium* collection, including proper techniques for collecting, drying, pressing, and mounting plants onto paper. Linnaeus also emphasized the relevance of collecting closely related specimens and using standardized collecting procedures to facilitate the exchange of materials and information among botanists. His efforts laid the foundations for the establishment of modern *herbarium* collections, which are still essential resources for botanical research and education [13]. In 1935, the International Association for Plant Taxonomy (IAPT) established the “Index *Herbarium*” (http://sweetgum.nybg.org/science/ih/), a comprehensive resource that serves as a global directory of *herbaria* from different independent contributors. Nowadays, it includes almost 400 million specimens provided by more than 3500 active *herbaria* worldwide [14]. Among them, the largest number of specimens are preserved at the “Muséum National d’Histoire Naturelle” (France), at the “New York Botanical Garden” (USA), at the “Komarov Botanical Institute” (Russia), and at the ”Royal Botanic Gardens” (UK) [14]. Until the mid-20th century, researchers had two options for examining *herbarium* collections: either by physically visiting the *herbaria* or by requesting specimens on loan for study. Towards the end of the 20th century, significant advances took place with the massive process of digitalization of *herbarium* collections [15], which resulted in the development of virtual *herbaria*. Thus, *herbarium* specimens were available as high-resolution images linked to electronically associated notes [16,17] that are accessible through online platforms. Such databases improved the exploitation and the exchange of plant specimens, facilitating collaborative research and conservation efforts and providing a powerful source for investigating plant diversity and evolution dynamics. However, as discussed below, aDNA can be fragmented, damaged, and present in minute amounts, posing several challenges associated with its exploitation for genomics studies.

### 1.2. Herbarium Genomics

In recent decades, paleogenomics has greatly benefited from significant advances in the field of molecular biology [18], including those in DNA extraction and amplification procedures and improvements in next-generation sequencing (NGS) strategies. This has enabled researchers to obtain molecular markers as well as whole-genome sequences (WGS) from specimens dating back several centuries, including those preserved in *herbaria*. Paleogenomics studies were also applied to the analysis of pathogens preserved within these specimens, which allowed detailed explorations of temporal signals of divergence among modern and ancient strains [19,20]. As stated above, the characterization and digitization processes of collections have greatly promoted the accessibility and utilization of ancient samples in a wide range of studies [15]. Traditionally, *herbarium* specimens have been employed for taxonomy and systematics studies. These collections still represent a significant resource for both the discovery and formal description of plant taxa. Moreover, molecular approaches have revolutionized the study of taxonomy, bringing significant advancements in resolving ambiguous phylogenetic relationships and classifications [21]. By exploiting aDNA from now-extinct species or ancient samples, researchers can gain valuable insights into the evolutionary history of different organisms and species and bridge gaps in our understanding of past biodiversity and present genetic diversity, also allowing for the reconstruction of phylogenetic trees [21]. Moreover, taking into consideration that the dissemination of species in novel geographic areas is associated with molecular adaptative mechanisms as well as significant phenotypic and phenological changes [22,23], *herbaria* can be exploited to dissect the plant response to changing environmental conditions across time [24,25]. Indeed, recruiting phenotypic and genotypic information from historical materials collected in different geographical areas is crucial to reconstructing the evolutionary dynamics and adaptation processes of a species. For example, Myers et al. (2022) [26] analyzed phenotypic data acquired from illustrations of the common bean from the XVI century to provide historical support to the dynamics of the introduction of the two main gene pools of this species (i.e., Mesoamerican and Andean) from the Americas to Europe following the travels of Columbus. The application of molecular tools to ancient biological samples might provide the opportunity to investigate population dynamics across different time points as a result of migration processes, gene flow, occurrence of novel mutations, and changes at the genome level also driven by genetic drift and by natural selection, although limitations can arise depending on the quality of the aDNA. This makes it possible to investigate the complex interplay between genetics, environment, and evolution of a population across time [27,28]. In genomic studies, the comparison of data from *herbaria* and modern samples can provide valuable information for optimizing the management of plant genetic resources [29]. Cozzolino et al. (2007) [30] evaluated changes in the genetic diversity of the *Anacamptis palustris* population because of habitat transformation in Italy over time. To do so, authors sequenced and compared a plastid region between *herbarium* specimens collected prior to the Second World War and modern samples. Thus, this approach could be very useful as a complement to large genotyping and phenotyping programs of modern accessions aimed at understanding the environmental adaptation and the evolutionary dynamics of genetic diversity, such as the INCRESE project [31], which is focused on food legumes such as chickpeas [32], the common bean [33], lupin [34], and lentil [35]. However, from the work carried out by Cozzolino et al. (2007) [30], some possible limitations of complementing modern collections with *herbarium* collections have arisen due to the limited sample size (i.e., the number of specimens) and potential methodological biases in collecting procedures. For instance, only a few individuals could have been collected from each collection site, which may not capture the full extent of genetic diversity within a population. Despite these limitations, *herbaria* can be considered a valuable source for detecting variations in the distribution and frequency of haplotypes and alleles.

### 1.3. Challenges in the Use of Herbaria and Ancient Samples for Genomics Studies

The advent of NGS platforms has opened unprecedented possibilities for studying genome-level diversity from *herbaria* and ancient samples. However, for this purpose, the successful implementation of NGS may be challenging due to the unique nature and the issues associated with the manipulation of ancient samples, such as damage and fragmentation of the aDNA. The development of robust protocols for aDNA purification, amplification, and sequence data analysis is crucial for obtaining reliable results [18]. The aDNA from *herbaria* and ancient samples is often degraded and fragmented, and it is often challenging to purify fragments longer than 500 bp [36,37,38]; thus, the optimization of extraction protocols can represent a crucial bottleneck in obtaining high-quality aDNA suitable for sequencing. Additionally, the limited amount of aDNA that can be rescued from ancient specimens poses the risk of contamination between samples and from modern DNA while handling and processing [39,40]. Moreover, aDNA in *herbarium*-preserved samples can be susceptible to biochemical postmortem damage [41]. Indeed, over time, the aDNA molecules can undergo various forms of degradation, including fragmentation and chemical modifications; these damages pose additional challenges when the original DNA sequence must be assembled and in the analysis of sequences. The biochemical postmortem damage in *herbarium* samples can result from different factors, such as light exposure, not-controlled moisture, temperature fluctuations, and the presence of reactive chemicals. These factors can lead to DNA strand breaks, base modifications, and crosslinking of DNA molecules, which can affect the quality and integrity of the assembled sequence [42]. Efficient protocols have been developed to overcome these issues, especially regarding aDNA purification. Here, we report the most relevant protocols for aDNA purification from *herbaria* specimens (Table 1). Furthermore, the choice of the bioinformatics pipeline and of the strategy for the analysis of NGS data from *herbaria* and ancient samples is particularly relevant, especially in the case of low-quality and fragmented aDNA. In the present review, we want to give insight into the best strategies for the extraction, purification, amplification, and sequencing of *herbarium* aDNA, which can be exploited in genetic diversity and evolutionary studies of plant species as well as in the reconstruction of phylogenetic and taxonomic relationships.

**Table 1:** Summary of relevant aDNA purification protocols from *herbaria* specimens. The source of plant material, timing of the sampling before storage, success with the tested samples, procedures for the evaluation of aDNA quality, and the reference are provided for each of the tested protocols.

DNA Extraction/Purification Protocol	Source of Plant Material	Timing of Sampling	Suitable for Extracting *Herbarium* DNA	Quality Evaluation Approach	Reference
CTAB, according to the protocol of Doyle and Doyle (1990)	*Juncus* and *Luzula genera (Juncaceae)*	*	Yes	PCR amplification	[43]
CTAB + pre-wash with a sorbitol-containing buffer	*Lafoensia* spp.	N.A.	Yes	PCR amplification	[44]
Modified CTAB	*Agropyronjunceum* (Gramineae), *Poa juncifolia* (Gramineae), *Poa palustris, Triticum aestivum* (Gramineae), *Vicia faba* (Fabaceae), *Zea mays* ssp. *mays*	N.A.	Yes	Restriction enzymes	[45]
	*Juncus* and *Luzula genera (Juncaceae)*	N.A.	Yes, but may present CTAB contamination	PCR amplification	[43]
	N.A.	≥60 years	Yes	PCR amplification	[46]
	Species from nine genera of the Papilionoideae	N.A.	Yes	PCR amplification	[47]
DNeasy Plant Mini Kit (QIAGEN)	*Juncus* and *Luzula genera (Juncaceae)*	N.A.	Yes	PCR amplification	[43]
DNA extraction with phenol purification and liquid nitrogen	*Juncus* and *Luzula genera (Juncaceae)*	N.A.	No	PCR amplification	[43]
Long-term precipitation in isopropanol and CsCl gradient	*Juncus* and *Luzula genera (Juncaceae)*	N.A.	No	PCR amplification	[43]
Proteinase K and sodium dodecyl sulfate (SDS)	*Scripus hattorianus*	1934	Yes	PCR amplification	[48]
N-phenacylthiazolium bromide (PTB)—dithiothreitol (DTT)	*Arabidopsis thaliana*	Between 1839 and 1898	Yes	NGS	[49]
Phenol-chloroform and silica spin column purification	*Herbarium* grape leaf tissue (unpublished data)	N.A.	Yes	PCR amplification	[50]
Polyvinylpyrrolidone PVP	genus *Dalbergia*	N.A.	Yes	PCR amplification	[51]
AMPure XP magnetic beads/PEG 8000-containing buffer	genus *Scorzonera*	Between 1920 and 1960	Yes	PCR amplification	[52]

* Modern plant samples that have been dried using sheets of paper to simulate *herbarium* specimen preparation. N.A.: not available.

## 2. Extraction and Purification of aDNA from *Herbarium* Specimens

The utilization of *herbarium* specimens in molecular studies poses significant challenges due to the difficulties in obtaining a substantial amount of high-quality purified aDNA [53]. Working with *herbarium* specimens involves the handling of a limited amount of tissues that are often stored under suboptimal conditions for long periods, leading to aDNA damage and an increased risk of contamination [43]. Doyle and Dickson (1987) [54] raised the importance of testing different methods to preserve aDNA integrity within *herbarium* specimens, and several subsequent studies have consistently found that air-drying of plant material is a reliable preservation method, preventing degradation and better preserving aDNA when compared to alternative practices, such as microwaving, boiling, or immersion in chemical solutions [37]. Moreover, it has been observed that well-preserved older materials tend to yield higher quantities of purified aDNA of better quality when compared to younger materials that have been subjected to unfavorable storage conditions [38,43,54]. As a general consideration, we also have to take into account that the initial condition of the tissues at the sampling stage, before storage, inevitably impacts the quality and the quantity of the purified aDNA [55]. Overall, the purification from *herbarium* specimens results in a lower yield of DNA when compared to the utilization of fresh plant tissues [4,56]. In their pioneering work, Rogers and Bendich (1985) [45] employed a modified CTAB (cetyltrimethylammonium bromide) nucleic acid extraction protocol to purify DNA from a small number of mummified seeds, *herbarium* specimens, and fresh plant tissues. Although this extraction method confirmed a higher yield when applied to fresh tissue, it has also proven to be effective for the extraction and purification of aDNA from ancient plant materials. A relevant step during aDNA purification is the grinding of the plant material obtained from the *herbarium* specimen. One of the most adopted practices relies on bead-based homogenization equipment, such as a mixer mill or bead mill. As noted by Drábková (2014) [57], this approach can reduce aDNA loss and the risk of contamination compared to alternative methods, such as grinding in mortars with liquid nitrogen. Additionally, the use of a bead-based homogenization approach enables the simultaneous processing of multiple samples, ensuring consistent and homogenous results [43]. It is important to remember that best practices must always be adopted in the laboratory to prevent contamination since the use of bead-based grinding methods might introduce a risk of contamination, which can particularly impact the analysis of aDNA. Over the past two decades, numerous aDNA extraction protocols have been developed, tested, and compared to optimize the quality and quantity of purified aDNA from *herbaria* specimens, for which a report is provided in Table 1. Drabkova et al. (2002) [43] tested seven DNA extraction protocols in *herbarium* specimens from *Juncus* and *Luzula* genera (*Juncaceae*) species collected during the twentieth century, including fresh tissue materials as a control. They evaluated quality parameters, such as the aDNA A260/280 and A230/280 absorbance ratios, and estimated the quantity of aDNA through spectrometry. From their results, they concluded that the use of a DNeasy Plant Kit (Qiagen), with some modifications, was the best approach for the aDNA purification. In detail, the authors found that both optimal homogenization of the plant tissue and extension of the precipitation time were crucial steps. Modifications to the manufacturer’s protocol included a longer cell lysis time (i.e., 30 min), an increased volume of AP1 buffer (i.e., 450 μL), a reduced volume of elution buffer (i.e., 50 μL) that increased aDNA concentration, and an extended elution time (i.e., 10 min). They also pointed out the protocols that gave the worst results, particularly the phenol extraction due to the potential contamination of aDNA with phenol, which can negatively affect downstream reactions, including amplification and sequencing, and, similarly, the CsCl gradients protocol, as aDNA could be lost within the gradient due to the limited amount of tissue [43].

Preserving the integrity of museum collections, which prioritizes maintaining specimens in their original state [58], poses a significant challenge when extracting DNA from ancient materials. To address this issue, efficient DNA extraction protocols that use minimal tissue have been established for historical specimens. Shepherd (2017) [59] introduced a non-invasive approach to sampling material directly from *herbaria* by using a Staedtler “Mars Plastic” eraser that minimized damage to the specimens while obtaining the necessary amount of tissue for DNA extraction. Sugita et al. (2020) [48], taking as an example a previously published protocol for DNA extraction in arthropods, established a plant-suitable non-disruptive protocol, particularly useful in species with small (i.e., <25 mm^2^) and fragile leaves [60,61]. Nevertheless, despite the importance of non-disruptive methods, CTAB and modified DNeasy Plant Mini Kit (QIAGEN) protocols are the most frequently adopted methods for aDNA extraction [57]. Among the reasons for this, aDNA-specific protocols are generally more expensive, more time-consuming, and require specific protocols and facilities to avoid contamination. As stated above, storage conditions for *herbarium* specimens (e.g., fluctuating temperatures or high humidity) and the species from which the tissue is obtained can influence the efficiency of the aDNA extraction [62]; thus, such factors must be taken into account when choosing the aDNA extraction protocol. With this regard, Marinček et al. (2022) [63] compared the standard Qiagen DNeasy Plant Mini Kit and a specific dithiothreitol (PTB)-dithiothreitol (DTT) protocol, previously developed by Gutaker et al. (2017) [49], with the aim of evaluating the efficiency of these protocols on specimens from the genera *Xanthium L.* and *Salix L.* They showed a lower efficiency of the Qiagen DNeasy Plant Mini Kit when working with older specimens (i.e., collected before 1900). Thus, a researcher might want to consider the PTB-DTT protocol as an alternative for aDNA extraction in older and more challenging specimens, for example, due to the higher concentration of secondary metabolites in the tissue. Indeed, several protocols have been developed, specifically taking into account the issues related to the presence of secondary metabolites that can inhibit PCR reactions. Ribeiro and Lovato (2007) [51] tested five DNA extraction protocols on fresh and *herbarium* leaves of various species belonging to the genus *Dalbergia*, known to contain significant amounts of secondary metabolites potentially interfering with DNA amplification. Based on their results, the most efficient protocol was developed by Jobes et al. (1995) [64]. This protocol uses three key reagents: polyvinylpyrrolidone (PVP), which binds phenolic compounds, sodium chloride with a high molar concentration, which inhibits the co-precipitation of polysaccharides and DNA and, in turn, enhances the solubility of polysaccharides in ethanol, and, finally, lithium chloride, which is useful to selectively precipitate and subsequently remove RNA. Krinitsina et al. (2015) [52] proposed a cost-effective DNA extraction method specifically designed for *herbarium* specimens that involves the utilization of AMPure XP magnetic beads diluted in a buffer containing PEG 8000. The authors suggest that the use of magnetic beads can reduce the concentration of PCR inhibitors in the aDNA. Hofreiter (2012) [65] adapted a protocol that had been originally designed for isolating aDNA from human hair samples [66] to ancient plant specimens. In particular, the protocol includes two key steps: a phenol–chloroform extraction followed by a silica spin column purification. Phenol–chloroform extraction has proven to be effective for those samples containing low amounts of DNA and that may contain polymerase chain reaction (PCR) inhibitors, affecting the enzymatic activity of the Taq polymerase [38,67]; although, one may consider minimizing the use of phenol-chloroform due to the environmental hazards associated with its use. However, the use of phenol–chloroform for DNA extraction can introduce contamination issues associated with phenol–chloroform residues that can significantly impact downstream processes, particularly DNA quantification and PCR, potentially compromising data accuracy [43].

**Figure 2:**
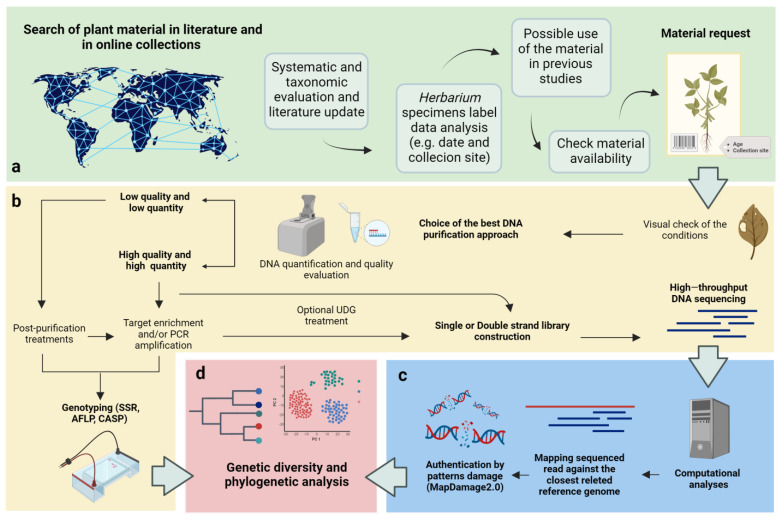
Workflow for the exploitation of *herbarium* specimens for genomic studies. (**a**) A preliminary investigation allows for gaining a comprehensive understanding of the available information on *herbarium* specimens. The use of web tools, such as web *herbaria*, academic databases, and online libraries, allows for finding reliable and up-to-date sources and conducting proper research before making any material requests; (**b**) the identification and selection of the best protocol—aDNA extraction, purification, or genotyping—to obtain molecular information from a specimen should take into account the characteristics of the material, available resources, and research objectives; (**c**) bioinformatics approaches are needed for data processing; (**d**) maximizing the quality of sequencing data is of great importance, as it significantly enhances the chances of reconstructing the phylogeny, understanding the demographic history of a species, and identifying selection signatures in response to natural or human-driven selection. Created using BioRender.com.

Overall, selecting the most appropriate aDNA purification protocols involves careful consideration of factors such as preservation conditions and the specific goals of the research. By taking these factors into account, researchers can optimize their DNA purification methods and enhance the chances of obtaining high-quality aDNA for subsequent analysis (Figure 2).

## 3. DNA Amplification by PCR from *Herbarium* Specimens

**Figure 3:**
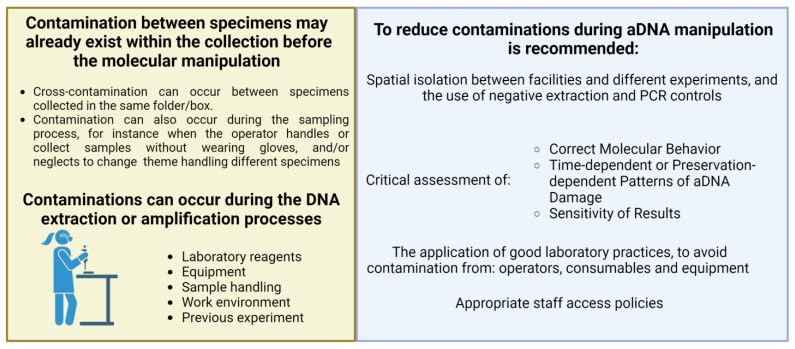
Contamination represents a challenge to acquire endogenous aDNA. Contamination sources and guidelines for minimizing negative impact during molecular manipulation of *herbarium* samples. Created using BioRender.com.

Since its development, PCR has become a routine and indispensable technique in several protocols for molecular biology studies that can be extended to the analysis of ancient samples. Several studies reported on the successful amplification of aDNA from plant *herbarium* material collected over a wide range of ages, resulting in variable lengths of amplified fragments [38,46,68]. Staats et al. (2011) [37] also reported that there are no significant differences in PCR amplification efficiency among plastid, mitochondrial, and nuclear aDNA from *herbarium* material, similar to what can be generally observed in amplification from plastid, mitochondrial, and nuclear DNA obtained from fresh tissue. As mentioned, DNA extraction from *herbarium* material can often yield low quantities of fragmented genetic material. However, the quality and purity, rather than the quantity, of the aDNA template mostly affect PCR amplification efficiency [38]. Quality control procedures, including rigorous purification steps and measures for minimizing and identifying contaminations, are crucial to ensuring the accuracy and reliability of the PCR amplification. As stated above, the presence of secondary metabolites in plant cells from fresh tissues, and thus in *herbarium* samples, can have a negative impact on the amplification efficiency. To address this issue, various extraction protocols have been developed with the aim of reducing the concentration of these metabolites [47,51,52]. In order to mitigate the effects of PCR inhibitors, as a post-aDNA purification step, a potential solution is diluting DNA extracts. However, this approach is not always feasible for *herbarium* samples due to the typically low yield and concentration of the eluted aDNA obtained from these specimens [69]. An alternative approach to improving the quality and quantity of the aDNA template available for PCR amplification in the presence of potential inhibitors has been suggested by Samarakoon et al. (2013) [69]; it involves the use of a reagent called TBT-PAR, which contains trehalose, bovine serum albumin (BSA), and polysorbate-20 (Tween-20). Multiple studies investigated the effects of using high concentrations of BSA on PCR amplification efficiency when working with poor-quality template aDNA. Overall, these studies agreed that a higher concentration of BSA can have a strong positive impact on PCR efficiency [51,57]. In particular, Särkinen et al. (2012) [38] suggested the utilization of a high concentration of BSA when routinely amplifying aDNA from *herbarium* specimens. Several factors can indeed affect PCR amplification efficiency, and among these factors are the purity and specific type of polymerase and the purity of buffers and reagents used. It is crucial to consider these factors to ensure reliable and consistent PCR results, especially in amplifying aDNA [70]. A suitable and cost-effective alternative to mitigate the effects of potential PCR inhibitors is the adoption of a polymerase enzyme that is less susceptible to their presence [71]. Särkinen et al. (2012) [38] suggest that the utilization of a polymerase without proofreading activity may perform better than one with proofreading activity. Moreover, it is essential to investigate and optimize the performance of different polymerases to improve the amplification efficiency for aDNA. Furthermore, Särkinen et al. (2012) [38] reported a significant negative correlation between the length of the aDNA template fragments from *herbarium* specimens and PCR efficiency, that is, a strong reduction in the success rate of the amplification when the template regions have a greater length than 100 bp. Thus, this suggests that shorter fragments can be easily amplified, which is an interesting aspect considering the fragmented nature of the aDNA template usually obtained from these specimens. However, in the case of extreme aDNA fragmentation, amplification of certain loci may be challenging. It is worth noting that mitochondrial and chloroplast aDNA can also be successfully retrieved from severely degraded samples, being present in high copy numbers within a cell. Moreover, there is a higher chance that some mtDNA and cpDNA fragments are long enough for amplification, even when nuclear DNA is highly fragmented [72]. Another relevant aspect is the instability of nucleic acids during long-term preservation, which can lead to the formation of various postmortem damages, affecting the quantity and quality of template aDNA for the amplification; such damages can also introduce potential errors that make the reconstruction of the aDNA sequence challenging. An example of postmortem damage is depurination, which can result in the formation of single- or double-strand breaks in the DNA molecule. Additionally, crosslinking reactions can occur, limiting or even preventing DNA amplification altogether [73]. Thus, it is crucial to identify the best methodologies and strategies to take into account the occurrence of postmortem damage when working with *herbarium* samples in order to mitigate their effects on quality and amplification efficiency. Indeed, oxidative and hydrolytic modifications of bases can lead to the formation of miscoding lesions, such as the deamination of cytosine to uracil. Thus, these lesions can lead to the incorporation of incorrect bases during the amplification process [37]. Certain oxidative damage can also create lesions that block the polymerase enzyme and promote the generation of chimeric sequences through ‘jumping PCR’ [41,74].To address the PCR artifact caused by misincorporation, Uracil-N-glycosylase (UNG) can be employed prior to amplification to remove deaminated cytosines [41,75]. UNG creates an abasic site that is subsequently hydrolyzed through β-elimination, resulting in a strand break [41,76]. However, it is important to carefully consider the use of UNG treatments since it inevitably leads to a reduction in the aDNA sequence length, which is often highly fragmented [77]. In addition to the sensitivity of PCR amplification, the low template aDNA concentration, and the fragmented nature of potential target loci in the aDNA, the presence of exogenous DNA contamination is also a common challenge in aDNA manipulation. Indeed, exogenous DNA might have been introduced during the collection and conservation of the plant material, by the presence of microorganisms or pathogens [39], or by handling samples during molecular processes. Kistler et al., 2020 [40] emphasized the importance of manipulating *herbarium* specimens in a physically isolated laboratory during all the steps. Nevertheless, DNA contaminants can still be introduced into the experimental workflow through various means, as summarized in Figure 3, including the use of contaminated reagents or samples as well as the presence of residual DNA and PCR amplification products from previous experiments [78].

To minimize the risk of contamination, Knapp et al. (2011) [79] developed a set of guidelines for establishing a molecular laboratory exclusively dedicated to studies on ancient specimens. The guidelines recommend conducting the different steps of aDNA extraction and amplification in dedicated rooms and/or hoods, expanding the concept of spatial isolation within the same laboratory. Furthermore, the authors highlight the importance of implementing a limited access policy, granting facility access only to qualified personnel who are aware of the risks of contamination.

## 4. Next-Generation Sequencing (NGS) and Genotyping on *Herbarium* Specimens to Disentangle Relevant Aspects of the Evolutionary History of a Species

Several studies investigated the genetic diversity preserved in herbarium collections, providing insights into relevant aspects of plant species’ evolutionary histories, including adaptation processes and the effects of environmental changes over time. Several approaches have been employed to genotype herbarium specimens [80,81,82]. Malenica et al. (2011) [83] successfully genotyped a 90-year-old Tribidrag grapevine herbarium specimen by utilizing a set of nine microsatellite markers (SSRs) and a whole-genome amplification (WGA) procedure. Other amplification-based genotyping approaches have proven to be suitable for molecular analysis of herbarium samples despite the limitations in the aDNA amplification from such materials, as described above [46,72]. As an example, while AFLP fingerprinting can be utilized to analyze herbarium material, the fragmented nature of the purified aDNA can introduce a bias when utilizing such an approach since AFLP is based on the presence and distribution of restriction sites. In such cases, Lambertini et al. (2008) [84] suggested using only those markers that can also be detected in fresh tissue and, in parallel, increasing the number of primer combinations to ensure the amplification of an adequate number of polymorphic fragments. By studying chloroplast and nuclear microsatellite diversity in 57 historical herbarium specimens, Roullier et al. (2013) [85] were able to trace relevant events in the evolutionary history of the sweet potato over time, such as the effect of the domestication process, migration patterns, and genetic interactions. By comparing the genetic data from historical herbarium collections with that of modern samples, researchers can assess changes in the level of genetic diversity and identify potential genetic bottlenecks or shifts that occurred during domestication and cultivation. The emergence of next-generation sequencing (NGS) led to a revolutionary change in our ability to obtain multi-locus genetic data from natural historical collections [72]. An approach that has significantly impacted this field of research is the Sequencing by Synthesis (SBS) strategy, which has been developed to enable cost-effective shotgun sequencing of whole genomes [86]. High-throughput SBS technologies involve the preparation of sequencing libraries, which entails attaching artificial DNA segments known as adapters to both ends of those template fragments characterized by a specific range of fragments [81]. This strategy allows for efficient and accurate sequencing of the aDNA fragments. When working with aDNA, differently from the SBS procedure for library preparation in modern samples, the fragmentation step that comes before adapter ligation can be skipped [81]. The adapters used in sequencing library preparation can serve multiple purposes. Indeed, they enable the priming of both whole-genome shotgun sequencing or the enrichment of specific genomic regions of interest through hybridization capture techniques [81]. These approaches allow for the characterization of various types of DNA, such as organellar DNA or nuclear loci, as well as the detection of a vast number of single-nucleotide polymorphisms (SNPs) distributed throughout the nuclear genome. Generally, library preparation protocols for *herbarium* DNA templates do not require significant modifications compared to standard ones. Most of these protocols were originally developed for Illumina sequencing-based platforms and can be broadly categorized into two main types: the single-stranded library (ss-library) and the double-stranded library (ds-library) construction methods. Briefly, ss-library preparation starts with heat denaturation of DNA followed by the attachment of a biotinylated adapter oligonucleotide to the 3′ ends. A second adapter is then blunt end-ligated to complete the library preparation, which is further amplified through PCR [87]. Whereas, ds-library preparation can be further categorized into Blunt-End and Y-adapter methods, both involving the end-repairing of the DNA fragments and ligation of double-strand adapters but differing in the type of adapter used. Bennett et al. (2014) [88] tested all the different methodologies for library preparation, starting from aDNA faunal and human remains, finding that the Y-adapter method led to the formation of adapter–dimer artifacts, while the ss-library approach allowed for an increased proportion of shorter endogenous molecules incorporated into the libraries. Their outcome has been further confirmed in recent research, suggesting that the ss-library preparation approach is better suited to the aDNA features compared to double-stranded library protocols [41], exhibiting greater sensitivity to degraded and poorly preserved ancient samples, such as *herbarium* specimens [89]. In protocols that require the amplification of libraries by PCR, the presence of artifacts caused by misincorporation can impact sequencing accuracy and efficiency. To mitigate the effects of postmortem damage, UDG enzyme-based protocols (see previous section) can be employed during library preparation [90]. One of the key advantages of using UDG is that it helps eliminate misincorporations (C to T and G to A) in the recovered sequences, improving the mapping efficiency. This is particularly useful when the reference sequence belongs to a species that is distantly related to the query sequence, which reduces the number of possible gaps and mismatches in the alignment [89]. However, the presence of uracil and other types of postmortem damage can also serve as an indicator to assess the presence of modern contamination in aDNA samples [91]. Damage profiles can be inferred using computational methods such as mapDamage 2.0, which allows for distinguishing ancient molecules from modern contaminants [92,93]. Indeed, the aDNA damage pattern is a crucial factor that can significantly impact aDNA analysis and the authentication of ancient sequences obtained from *herbarium* samples. Another strategy for addressing errors in sequence reconstruction resulting from misincorporations in aDNA is to focus downstream analysis on transversions rather than transitions since the latter are more susceptible to postmortem damage. Trucchi et al. (2021) [94] applied this strategy in their paleontological study of ancient bean seeds from different archaeological sites in Argentina. They analyzed the temporal dynamics of genetic variation and selection during the domestication process of the common bean (*Phaseolus vulgaris* L.) in the southern Andes by comparing WGS data from a panel of 15 ancient beans dating between 2500 and 600 years ago and modern wild and domesticated common bean accessions (from both Mesoamerican and Andean gene pools). The work of Trucchi et al. (2021) [94] clearly demonstrated how, by considering transversions, researchers can significantly reduce the impact of postmortem damage on sequence analysis and improve the accuracy of genetic variation inference in aDNA studies.

## 5. Conclusions

The employment of *herbaria* in paleogenomics can offer an opportunity for investigating the genetic and phenotypic diversities of ancient samples compared to modern ones, being available plant genetic resources that are largely unexplored. This requires optimized strategies for *herbaria* handling and for the analysis of the aDNA, including the employment of advanced next-generation sequencing approaches and computational methods to authenticate and analyze aDNA data and access an unexplored source of genetic information. However, the quality of attainable genetic data can be strongly affected by the protocols used for aDNA extraction, amplification, and sequencing due to several factors, such as the preservation state of the specimens and the aDNA. Consequently, an in-depth understanding of the strengths and limitations of available protocols for aDNA exploitation becomes crucial. Here, we reviewed the most recommended strategies and approaches for handling *herbaria* specimens and aDNA. Moreover, we discussed the relevance of integrating available high-quality ancient sequence data from *herbarium* specimens with modern sequences to perform population genetics and genomics studies and inferences on the evolution of a species.

## Data Availability

No new data were created or analyzed in this study. Data sharing is not applicable to this article.
